# Impact of comorbidities on COVID-19 mortality in hospitalized women: Insights from the metropolitan area of the Valley of Mexico from 2020 to 2022

**DOI:** 10.1016/j.ijregi.2024.100420

**Published:** 2024-08-08

**Authors:** Diego Francisco Benítez-Chao, Marisela García-Hernández, José M. Cuellar, Gabriel García, Jose Francisco Islas, Elsa N. Garza-Treviño, Gerardo R. Padilla-Rivas

**Affiliations:** Universidad Autónoma de Nuevo León, Facultad de Medicina, Departamento de Bioquímica y Medicina Molecular, Dr. Eduardo Aguirre Pequeño, Monterrey CP.64460, México

**Keywords:** Hospitalized women, Comorbidities, Mortality risk factors, Mexico City

## Abstract

**Objectives:**

This research summarizes the impact of the major comorbidities impacting hospitalized women with COVID-19 and their relation to death.

**Methods:**

Public data from national databases (2020-2022) for hospitalized women, including identification data, hospitalization time, comorbidities, and intensive care unit (ICU) admissions, were analyzed. Women were stratified by age (split at 50 years). Binary regression models determined the correlation between comorbidities and COVID-19 with mortality, expressed as odds ratios.

**Results:**

A total of 46,492 women were hospitalized, with 70.1% aged above 50 years. A total of 17,728 fatalities occurred, with 86.5% in the older age group. A total of 5.82% women required intensive care. The common comorbidities were pneumonia, hypertension, diabetes, obesity, and intubation. A total of 56.6% died within the 1^st^ week; in the ICU, 65.7% died by week 2. In the logistic regression, diabetes and chronic kidney disease (CKD) were initially significant, followed by pneumonia and CKD (days 8-14), intubation and, ICU stay (beyond the 15^th^ day). In the ICU, intubation impact worsened over time.

**Conclusions:**

Our study highlights the significant impact of comorbidities on COVID-19 mortality in women in the Valley of Mexico. Pneumonia, diabetes, CKD, and intubation were notably prevalent and correlated strongly with death in older women. Timely intubation improves survival, whereas delayed intubation increases mortality risk, particularly, in the ICU. Urgent targeted interventions are required, especially for older hospitalized women.

## Highlights

• Nearly 38% of hospitalized women with COVID-19 died.
• The major comorbidities were pneumonia, hypertension, diabetes, obesity, and intubation.
• In the general wards, 56.6% died within the 1^st^ week; in the intensive care unit wards, 65.7% died during the 2^nd^ week.
• First week mortality risk factors were diabetes, pneumonia, and chronic kidney disease.
• The intensive care unit mortality drivers were intubation, chronic kidney disease, and pneumonia.

## Introduction

Mexico has the 10th largest population in the world and is the second largest country in North America, with around 126 million people. Mexico City and its surrounding metropolitan area, known as the Valley of Mexico, house approximately 21 million people, which accounts for almost 20% of the country's population. This makes the Valley of Mexico one of the largest urban areas in the world [1]. Interestingly, given the number of residents, the Valley of Mexico represents approximately 4% of the total Latin American population, with a demographic makeup comprising 54.14% women, which is just slightly above the national average (52%), with 24% of these women aged 50 years and older, 46% aged between 20 and 49 years, and 30% below 20 years [2]. Given that women makeup over half of the population of the Valley of Mexico, prioritizing health care becomes a critical step for the advancement of the community. Thus, ensuring access to comprehensive health services, including reproductive health care and preventive screenings, becomes critical for meeting the diverse health requirements of women of all ages [3,4].

Through the natural aging process, women between the ages of 45 and 55 years experience a natural hormonal decline. This transition time involves biological modifications, particularly, related to their health and well-being [5]. This pivotal stage in a woman's life increases the need for more thorough examination to best understand the individual needs of each woman. Vasomotor symptoms, including hot flashes and night sweats, represent common manifestations of this hormonal transition, affecting quality of life and daily functioning [6]. Metabolic disorders, such as hypertension, dyslipidemia, diabetes, and abdominal obesity, are higher in menopausal and postmenopausal women and, therefore, are at a potential heightened risk of cardiovascular diseases (CVDs) [5,7]. This middle-aged state leads to an increase in the prevalence of metabolic syndrome. Metabolic syndrome is a collection of malignancies, including hyperinsulinemia, insulin resistance, and intra-abdominal fat, all of which may contribute to dyslipidemia, inflammation, and atherosclerosis, significantly impacting overall health and quality of life [5]. Hence, investing in women's health yields profound benefits not only at the individual level but also at the society level because healthy women are better positioned to participate in the workforce, pursue education, and foster resilience against health challenges.

To enhance our understanding of the major factors influencing health outcomes, we sought to delineate the prevalence of the major comorbidities which played a critical role in hospitalized women with COVID-19 spanning from 2020 to 2022. Specifically, we aimed to describe the distribution of such comorbidities across two distinct age groups of women: those under 50 years old (younger) and those 50 years and above (older); the latter being recognized as a vulnerable population because of the common presence of chronic health conditions. Furthermore, we related the influence of comorbidities within both age groups and the hospitalization time spent in the deceased patients with COVID-19.

## Methods

Publicly available data were obtained from “*Casos a nivel nacional asociados a* COVID-19,” a national open public health database provided by the Mexico City's Ministry of Health (https://datos.cdmx.gob.mx/dataset/casos-asociados-a-covid-19) [8]. This database contains nearly 6 million entries from the national public health sector from 2020 to 2022. From the database, we filtered the entries to include only hospitalized women with a positive diagnosis for COVID-19, giving a final count of 46,492, from which 32,597 (70.1%) were from women aged 50 years or older.

Women, due to the natural aging process, tend to experience hormonal decline around 50 years [9]. Based on this natural occurrence, we divided our study population into two groups: those younger than 50 years old and those 50 years old and older.

To identify positive COVID-19 cases and given the database structure, we filtered cases containing the following terms: “*CASO DE* SARS-COV-2 *CONFIRMADO*” (confirmed SARS-CoV-2 case), “*CASO DE* COVID-19 *CONFIRMADO POR ASOCIACIÓN CLÍNICA EPIDEMIOLÓGICA*” (confirmed COVID-19 case by epidemiological clinical association), and “*CASO DE* COVID-19 *CONFIRMADO POR COMITÉ DE DICTAMINA*CIÓN” (confirmed COVID-19 case by medical committee review). Any cases not containing these terms were excluded.

It is important to denote that the national database holds information on age; year of entry; gender; decease date; and comorbidities including diabetes, pneumonia, chronic obstructive pulmonary disease, asthma, immunosuppression, hypertension, CVDs, obesity, renal failure, and other risk factors. The database shows whether admitted patients required intensive care unit (ICU) attention and an intubation procedure. All national registry data includes a patient registry number, which does not link any entry to any patient information, as directed under federal data protection law “*Ley de la protección de datos personales en posesión de sujetos obligados*” (https://www.diputados.gob.mx/LeyesBiblio/pdf/LGPDPPSO.pdf) [10] to help protect patient anonymity.

### Frequencies and relation to deaths by groups and comorbidities

We recorded the frequency for all comorbidities according to the registry; age was set in two groups: young women (aged under 50 years) and older women (aged 50 years and above). Data were presented using ICU admission or death as a limiting parameter.

### Frequencies of admission to death

We further determined the time between admission to death or hospitalization time in critical time intervals of 0-3 days, 4-7 days, 8-14 days, and more than 15 days.

### Binary logistic regression analysis

Both age groups were analyzed through a binary logistic regression to determine the influence of each studied comorbidity. Significant values (*P* <0.05) were re-analyzed via a second logistic regression. Analyzes were established using SPSS Statistics (version 23.0) (IBM Corp., Armonk, NY, USA).

## Results

### General women's population statistics

This study included a total of 46,492 women who tested positive for COVID-19 who were admitted to the Mexican public health care system from 2020 to 2022. After collecting the initial data, we categorized the age range into two populations: individuals under 50 years (younger) and those above 50 years (older). Of this population, 13,894 (29.9%) cases were younger women and 32,597 (70.1%) cases were older women (Table 1).

**Table 1:** General profile of positive hospitalized women in the Mexican health care system.

Characteristics		<50 years old		>50 years old		Total population			<50 years old		>50 years old		Total population			<50 years old		>50 years old		Total population		
		n	(%)	n	(%)	N	(%)	*P*-value	n	(%)	n	(%)	N	(%)	*P*-value	n	(%)	n	(%)	N	(%)	*P*-value
Total		13,894	29.9	32,597	70.1	46,492	100.0		820	30.3	1886	69.7	2706	100.0		2394	13.5	15,334	86.5	17,728	100.0	
Intubated	yes	1299	9.3	4947	15.2	6246	13.4	**<0.001**	515	62.8	1467	77.8	1982	26.8	**<0.001**	857	35.8	4124	26.9	4981	28.1	**<0.001**
	no	12,595	90.7	27,650	84.8	40,245	86.6		305	37.2	419	22.2	724	73.2		1537	64.2	11,220	73.1	12,758	71.9	
Diabetes	yes	1899	13.7	11,527	35.4	13,427	28.9	**<0.001**	133	16.2	651	34.5	784	29.0	**<0.001**	572	23.9	5760	37.5	6333	35.7	**<0.001**
	no	11,995	86.3	21,070	64.6	33,065	71.1		687	83.8	1235	65.5	1922	71.0		1822	76.1	9584	62.5	11,406	64.3	
Pneumonia	Yes	7112	51.2	21,852	67.0	28,964	62.3	**<0.001**	644	78.5	1733	91.9	2377	87.8	**<0.001**	1799	75.1	11,252	73.3	13,051	26.4	0.062
	no	6782	48.8	10,745	33.0	17,528	37.7		176	21.5	153	8.1	329	12.2		595	24.9	4092	26.7	4688	73.6	
Chronic obstructive pulmonary disease	yes	101	0.7	1553	4.8	1654	3.6	**<0.001**	5	0.6	78	4.1	83	3.1	**<0.001**	27	1.1	822	5.4	849	4.8	**<0.001**
	no	13,793	99.3	31,044	95.2	44,838	96.4		815	99.4	1808	95.9	2,623	96.9		2,367	98.9	14,522	94.6	16,890	95.2	
Asthma	yes	385	2.8	630	1.9	1015	2.2	**<0.001**	15	1.8	34	1.8	49	1.8	**<0.001**	69	2.9	240	1.6	309	1.7	**<0.001**
	no	13,509	97.2	31,967	98.1	45,477	97.8		805	98.2	1852	98.0	2657	98.2		2325	97.1	15,104	98.4	17,430	98.3	
Immunosuppression	yes	495	3.6	933	2.9	1428	3.1	**<0.001**	68	8.3	90	4.8	158	5.8	**<0.001**	118	4.9	432	2.8	550	3.1	**<0.001**
	no	13,399	96.4	31,664	97.1	45,064	96.9		752	91.7	1796	95.2	2548	94.2		2276	95.1	14,912	97.2	17,189	96.9	
Hypertension	yes	1775	12.8	14,513	44.5	16,288	35.0	**<0.001**	115	14.0	836	44.3	951	35.1	**<0.001**	543	22.7	7324	47.7	7,867	44.3	**<0.001**
	no	12,119	87.2	18,084	55.5	30,204	65.0		705	86.0	1060	55.7	1755	64.9		1851	77.3	8020	52.3	9872	55.7	
Cardiovascular disease	yes	241	1.7	1664	5.1	1905	4.1	**<0.001**	24	2.9	136	7.2	160	5.9	**<0.001**	57	2.4	812	5.3	869	4.9	**<0.001**
	no	13,653	98.3	30,933	94.9	44,587	95.6		796	97.1	1750	92.8	2546	94.1		2337	97.6	14,532	94.7	16,870	95.1	
Obesity	yes	2763	19.9	6994	21.5	9757	21.0	**<0.001**	213	26.0	469	24.9	682	25.2	0.583	660	27.6	3313	21.6	3973	22.4	**<0.001**
	no	11,131	80.1	25,603	78.5	36,735	79.0		607	74.0	1417	75.1	2024	74.8		1734	72.4	12,031	78.4	13,766	77.6	
Chronic kidney disease	yes	490	3.5	1626	5.0	2116	4.6	**<0.001**	38	4.6	97	5.1	135	5.0	0.631	193	8.1	962	6.3	1155	6.5	**<0.001**
	no	13,404	96.5	30,971	95.0	44,376	95.4		782	95.4	1789	94.9	2571	95.0		2201	91.9	14,382	93.7	16,584	93.5	
Smoker	yes	777	5.6	1742	5.3	2519	5.4	0.283	35	4.3	100	5.3	135	5.0	0.291	142	5.9	804	5.2	946	5.3	0.169
	no	13,117	94.4	30,855	94.7	43,973	94.6		785	95.7	1786	94.7	2571	95.0		2252	94.1	14,540	94.8	16,793	94.7	
Intensive care unit	yes	820	5.9	1886	5.8	2706	5.8	0.635								310	12.9	1273	8.3	1583	8.9	**<0.001**
	no	13,074	94.1	30,711	94.2	43,786	94.2									2084	87.1	14,071	91.7	16,156	91.1	

Bold numbers mean significance.

**Figure 1:**
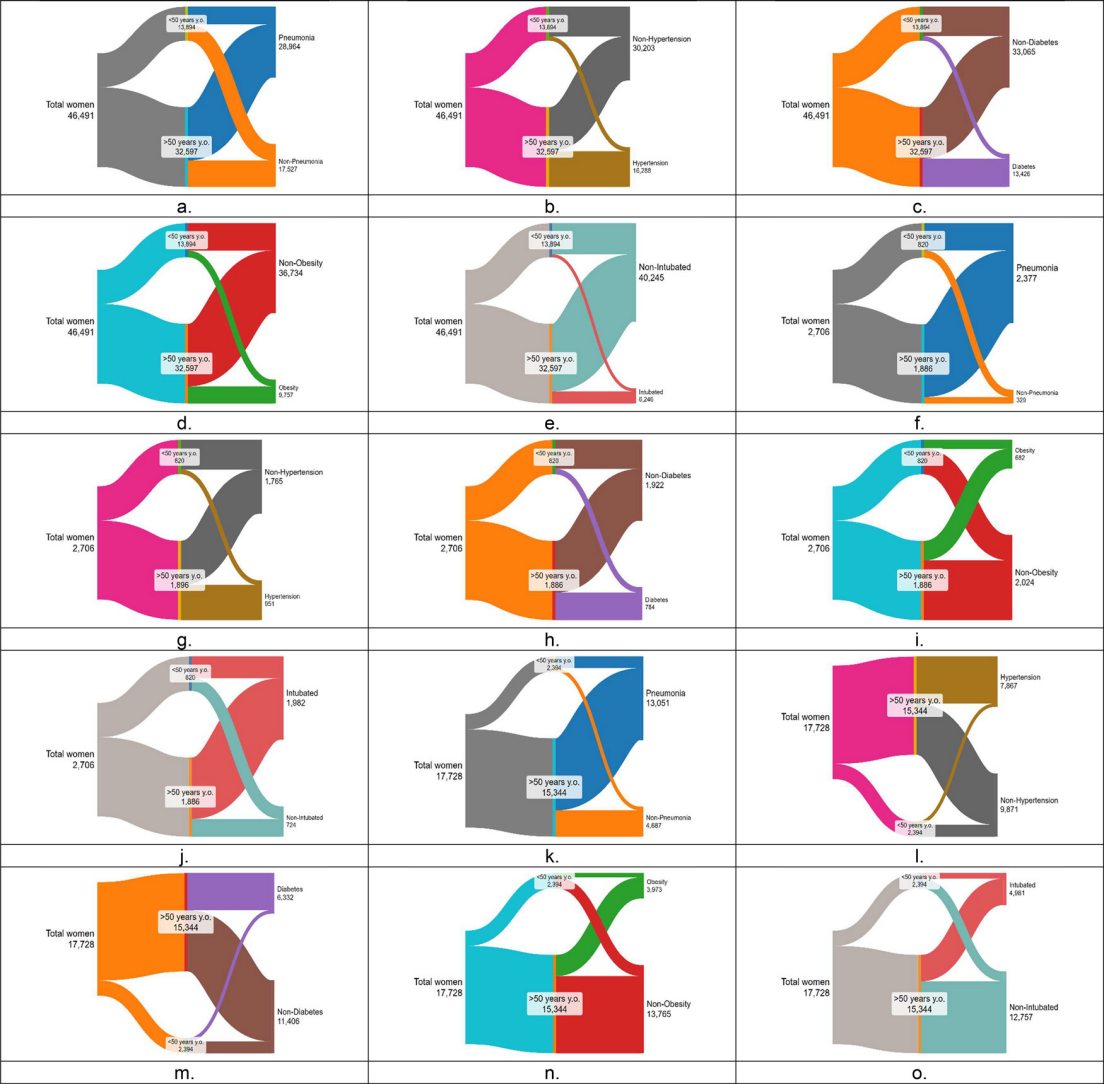
(a-o). Distribution of the total cases among female patients across two age groups (below 50 years and over 50 years) for (a-e) hospital patients with (a) pneumonia, (b) hypertension, (c) diabetes, (d) obesity, and (e) intubation; (f-j) intensive care unit admissions with (f) pneumonia, (g) hypertension, (h) diabetes, (i) obesity, and (j) intubation; (k-o) total deaths with (k) pneumonia, (l) hypertension, (m) diabetes, (n) obesity, and (o) intubation. y.o.= years old.

Our initial assessment detected five major comorbidities affecting both female populations, as seen in Figure 1 (a-e), which are present in over 10% of all patients. Table 1 (total population) shows that the most common comorbidity was pneumonia, with 28,964 positive cases divided into 7112 or 51.2% of the younger population and 21,852 or 67.0% of the older population, followed by hypertension with 16,288 cases, with 1775 (12.8%) and 14,513 (44.5%) cases, respectively. Diabetes came in third place with 13,427 cases, from which 1899 (13.7%) cases were from the younger population and 11,527 (35.4%) cases were from the older population. Obesity followed with 9757 cases, with 2763 (19.9%) cases from the younger population and 6994 (21.5%) cases from the older population. Finally, intubated women came in fifth place with 6246 cases, from which 1299 cases (9.3%) were from the younger population and 4497 (15.2%) cases were from the older population (Table 1).

Table 1 (ICU population) and Figure 1 (f-j) display the total women population requiring intensive care. Critical cases admitted to the ICU accounted for 2706 (5.82%) cases of the total population, from which 1886 cases were for the older population. The findings from the ICU admissions showed a significant elevation in comorbidity rates in women from each group. Pneumonia was the most common comorbidity present in the ICU with 2377 cases, wherein 644 (78.5%) cases were from the younger population and 1733 (91.9%, almost all) cases were of the older population. In addition, we observed that nearly three-quarters of women in the ICU underwent intubation or a total of 1982 cases, of whom 515 (62.8%) patients were from the younger population and 1467 (77.8%) cases were from the older population. Interestingly, a third of all patients had hypertension or 951 cases, of whom 115 (14.0%) belonged to the younger women and 836 (44.3%) to the older women. Finally, other chronic conditions, such as diabetes with 651 cases from 784 total cases and obesity with 469 cases from 682 cases, were more prevalent in the older patient group.

### Relevance of comorbidities in death stats

Table 1 (death population) shows cases and comorbidities present in the deaths registered in the female population. Unfortunately, 17,732 cases ended in death; from which 2394 (13.5%) cases belonged to the younger population and 15,334 (86.5%) cases to the older population.

Of the top comorbidities in this deceased population, pneumonia had the highest prevalence, which was similar in both age groups because it was present in 75.1% (1799 cases) of the younger population and 73.3% (11,252 cases) of the older population. A more detailed comparison between the older and younger populations reveals distinct health trends. Older women exhibit higher rates of hypertension and diabetes than their younger counterparts. Specifically, 47.7% of older women have hypertension compared with only 22.7% of the younger population. Similarly, diabetes affects 37.5% (5760 cases) of older women compared with 23.9% (572 cases) of the younger group. In contrast, the younger population shows higher rates of intubation and obesity. Intubation was seen in 35.8% (857 cases) of the younger group, whereas only 26.9% (4124 cases) of older women require it. Likewise, obesity was more prevalent in the younger population, with 27.6% (660 cases) affected compared with 21.9% (3313 cases) of older women.

The duration of hospital stays (Table 2) was classified into four segments: 0-3 days, 4-7 days, 8-14 days, and more than 15 days. Of the general hospitalized population, 56.6% or 10,036 women died within the 1^st^ week. A more in-depth analysis showed that from those admitted to ICU, 34.7% (550 cases) died within the 1^st^ week. The most affected patients were of the older population because they had the highest incidence of mortality at all different time intervals, representing 86.5% (1533 cases) of deaths in the hospitalized population and 80.4% (1273 cases) in the ICU population.

**Table 2:** Hospitalization duration until death for female patients, including intensive care unit admissions.

Population type	Admission-death (days)	Under 50 years old		50 years old and above		Total	
		n	(%)	n	(%)	n	(%)
Hospitalized women with COVID-19	0-3 days	754	31.5	4782	31.2	5537	31.2
	4-7 days	601	25.1	3899	25.4	4501	25.4
	8-14 days	612	25.6	4020	26.2	4633	26.2
	>15 days	427	17.8	2633	17.2	3061	17.2
	Total	2394	13.5	15,334	86.5	17,728	100
Intensive care unit hospitalized women with COVID-19	0-3 days	63	20.3	211	16.6	274	16.6
	4-7 days	50	16.1	226	17.8	276	17.8
	8-14 days	90	29	351	27.6	441	27.6
	>15 days	107	34.5	485	38.1	592	38.1
	Total	310	19.6	1273	80.4	1583	100

### Individual comorbidity analysis

In the assessment, a complete panorama of both populations’ binary logistic regression models was developed, taking into consideration all comorbidities. The distribution of top comorbidities present in ICU-admitted patients who died are displayed in Figure 1 (k-o). Binary logistic regression was used to evaluate the impact of the individual comorbidities in deceased women across varying hospitalization duration, as shown in Table 3a. Those admitted to the ICU are shown in Table 3b. Statistically significant variables were identified in the general women population who inevitably died during their hospital stay (n = 17,728) within 0-3 days of admission, with diabetes (odds ratio [OR]: 1.124) and chronic kidney disease (CKD) (OR: 1.189) exhibiting significance. In the 4- to 7-day period, pneumonia (OR: 1.105) and CKD (OR: 1.146) emerged as the most influential variables. For the 8- to 14-day period, intubation (OR: 1.232) and immunosuppression (OR: 1.212) were identified as significant factors. Of those who died 15 days or more after admission, the most significant variables were intubation (OR: 1.855) and ICU admission (OR: 2.210).

**Table 3a:** Binary logistic regression analysis of hospitalized women in general wards.

All deaths of hospitalized women							All deaths of hospitalized women aged 50 years and over							All deaths of hospitalized women aged under 50 years						
0-3 days	B	SE	Wald	DF	*P*-values	OR	0-3 days	B	SE	Wald	DF	*P*-values	OR	0-3 days	B	SE	Wald	DF	*P*-values	OR
DIAB	0.116	0.035	11.340	1	0.001	1.124	DIAB	0.088	0.037	5.639	1	0.05	1.092	DIAB	0.395	0.101	15.280	1	p<0.001	1.485
CKD	0.173	0.066	6.961	1	0.05	1.189	CKD	0.161	0.072	5.038	1	0.05	1.175							
4-7 days	B	SE	Wald	DF	*P*-values	OR	4-7 days	B	SE	Wald	DF	*P*-values	OR	4-7 days	B	SE	Wald	DF	*P*-values	OR
PNA	0.099	0.040	6.114	1	0.05	1.105	PNA	0.107	0.043	6.132	1	0.05	1.113	CVD	0.629	0.281	4.989	1	0.05	1.875
CKD	0.136	0.068	3.984	1	0.05	1.146	CKD	0.163	0.074	4.774	1	0.05	1.177							
8-14 days	B	SE	Wald	DF	*P*-values	OR	8-14 days	B	SE	Wald	DF	*P*-values	OR	8-14 days	B	SE	Wald	DF	*P*-values	OR
INT	0.208	0.037	31.135	1	0.001	1.232	INT	0.214	0.041	27.743	1	0.001	1.238	INT	0.197	0.097	4.173	1	0.05	1.218
IMMUNO	0.192	0.095	4.069	1	0.05	1.212	IMMUNO	0.290	0.105	7.597	1	0.05	1.337							
≥ 15 days	B	SE	Wald	DF	*P*-values	OR	≥ 15 days	B	SE	Wald	DF	*P*-values	OR	≥ 15 days	B	SE	Wald	DF	*P*-values	OR
INT	0.618	0.047	175.927	1	0.001	1.855	INT	0.633	0.050	157.653	1	0.001	1.884	INT	0.555	0.123	20.531	1	0.001	1.742
IMMUNO	0.793	0.064	155.429	1	0.001	2.210	ICU	0.815	0.070	135.384	1	0.001	2.260	IMMUNO	0.545	0.219	6.205	1	0.05	1.725
														ICU	0.708	0.152	21.737	1	0.001	2.030

**Table 3b:** Binary logistic regression analysis of hospitalized women in ICU wards.

All deaths of hospitalized women							All deaths of hospitalized women aged 50 years and over							All deaths of hospitalized women aged under 50 years						
0-3 days	B	SE	Wald	DF	*P*-values	OR	0-3 days	B	SE	Wald	DF	*P*-values	OR	0-3 days	B	SE	Wald	DF	*P*-values	OR
INT	0.556	0.164	11.429	1	0.001	1.743														
CKD	0.505	0.249	4.132	1	0.05	1.658														
4-7 days	B	SE	Wald	DF	*P*-values	OR	4-7 days	B	SE	Wald	DF	*P*-values	OR	4-7 days	B	SE	Wald	DF	*P*-values	OR
INT	0.947	0.202	21.986	1	0.001	2.577	CKD	0.801	0.258	9.655	1	0.05	2.228							
PNA	0.877	0.324	7.344	1	0.05	2.404	COPD	0.564	0.286	3.891	1	0.05	1.758							
CKD	0.911	0.220	17.137	1	0.001	2.487														
8-14 days	B	SE	Wald	DF	*P*-values	OR	8-14 days	B	SE	Wald	DF	*P*-values	OR	8-14 days	B	SE	Wald	DF	*P*-values	OR
INT	0.829	0.154	29.096	1	0.001	2.290	INT	1.112	0.231	23.124	1	0.001	3.039							
PNA	0.921	0.251	13.430	1	0.001	2.511	AA	1.417	0.544	6.783	1	0.05	4.124							
COPD	0.649	0.260	6.247	1	0.05	1.913														
≥ 15 days	B	SE	Wald	DF	*P*-values	OR	≥ 15 days	B	SE	Wald	DF	*P*-values	OR	≥ 15 days	B	SE	Wald	DF	*P*-values	OR
INT	2.077	0.199	108.595	1	0.001	7.983								INT	1.476	0.548	7.249	1	p<0.05	4.375
PNA	0.849	0.247	11.826	1	0.001	2.337														

AA, asthma; B, binary regression model; CVD, cardiovascular disease; CKD, chronic kidney disease; COPD, chronic obstructive pulmonary disease; DF, degrees of freedom; DIAB, diabetes; HT, hypertension; ICU, intensive care unit; IMMUNO, immunosuppression; INT, intubated; OR, odds ratio; PNA, pneumonia; SE, standard error; SMOKE, smoker.

For deceased young patients (Table 3a), diabetes (OR: 1.485) emerged as the most significant mortality risk factor within the first 3 days after admission to the hospital. Subsequently, from days 4 to 7, CVDs became significant (OR: 1.875). Then, intubation emerged as increasingly significant from day 7 onward, with the OR rising steadily from days 8 to 14 (OR: 1.218) and further from day 15 onward (OR: 1.742). For patients with prolonged hospital stays (i.e. 15 days or more), other significant factors present included immunosuppression (OR: 1.725) and ICU admission, which had the highest OR among all variables (OR: 2.030).

Regarding older patients who died (Table 3a), CKD emerged as a critical factor within the initial week of hospitalization. This was clear in patients who died during the first 0-3 days (OR: 1.175) or persisting until the end of the 1^st^ week (OR: 1.177). Other notable factors during this period included diabetes (OR: 1.092, 0-3 days) and pneumonia (OR: 1.113, 4-7 days). As for those who survived until the 2^nd^ week, intubation became the top factor, present for 8-14 days in the hospital (OR: 1.238) and increased in those admitted for 15 days or more (OR: 1.884). Notably, the OR for immunosuppressed patients during the 2^nd^ week (OR: 1.337) and for patients hospitalized with 15 or more days admission to the ICU, alongside intubation, had substantially increased (OR: 2.260).

Binary regression models were once again used to identify significant ratios in hospitalized women in the ICU (Table 3b). During the 1^st^ week after admission to the ICU, notable comorbidities in general deceased female patients included intubation and CKD, with both conditions progressively worsening (every 3 days, they were doubling their odds), as detailed in Table 3b. Survivors entering the 2^nd^ week in the ICU faced a different set of challenges, including pneumonia and intubation as the main mortality risk factors. For women who required intubation, their ORs surged from 2.290 at the beginning of this week to a concerning 7.983 for patients who stayed 15 days or more in the ICU (Table 3b).

Particularly, renal compromised older patients in the ICU faced high mortality odds, with an OR of 2.228 on the first 4-7 days after ICU admission (Table 3b). Moving into the 2^nd^ week, chronic respiratory conditions became predominant, with diseases such as chronic obstructive pulmonary disease and asthma emerging as significant factors, exhibiting ORs of 1.758 and 4.124, respectively. Interestingly, in younger women, intubation exhibited strong ORs (OR: 4.375) after 15 or more days, which were higher than those for older women (OR: 3.039).

## Discussion

In this study, we determined the prevalence of comorbidities in hospitalized women with COVID-19, with pneumonia, hypertension, diabetes, intubation, and obesity being the most significant. As expected, pneumonia was the most prevalent comorbidity in patients under these conditions [11]. Indeed, the prevalence of comorbidities was consistently higher in older women than their younger counterparts. Previous studies have reported that women who underwent age-related hormonal decline (related to the older age) faced heightened vulnerability to severe outcomes, particularly, in the presence of underlying health conditions, such as CVDs, obesity, and diabetes, requiring heightened vigilance and targeted interventions to mitigate COVID-19–related risks effectively [12,13]. Similarly, a study from our group related to cardiovascular health during the pandemic found that the most common health issues reported in hospitalized women with COVID-19 aged 20 to 60 years were high blood pressure (11.9%), obesity (9.59%), and diabetes (8.74%) [1].

In this study, the prevalence rates reported for diabetes and hypertension remained consistent across all age groups of hospitalized women, whether admitted to regular hospital wards or the ICU (Table 1). However, diabetes emerged as a significant mortality risk, particularly, within the first 3 days after admission, with older populations exhibiting an OR of 1.092 and younger women exhibiting even higher ratios (OR: 1.485, Table 3a). These findings add to existing knowledge by showing that women with diabetes with severe COVID-19 tend to have shorter survival times, reaching similar conclusions as those described by Balta Başı *et al.* [14]. Diabetes affects 10.3% of Mexico's population. Furthermore, diabetes is known to induce pro-inflammatory markers, such as cytokines, including several interleukins and tumor necrosis factor, by the activation of various biochemical parameters, such as C-reactive protein. Moreover, diabetes-related risk increases with age, from 21.9% in women aged 50 to 59 years to 35.6% in those aged 60 to 69 years, with a slight drop to 32.7% in those aged 70 years and older [15,16].

Between days 4 and 7 after admission, diabetes ceased to be the primary mortality risk factor, with CKD as a notably poor survival prognosis factor for COVID-19 hospitalized women, with a significantly increased OR (OR: 1.177) (Table 3a). These findings match others regarding the length of hospitalization time in adult patients with COVID-19 with advanced CKD and end-stage kidney disease, wherein death was seen within 10.5 days, reflecting the severity of their condition [17] Moreover, the survival prognosis for intubated hospitalized young women worsens with each day after the 2^nd^ week of admission. This trend is evident by the increase in OR, peaking at 1.884 during day 15 and over (Table 3a), suggesting an important association between prolonged duration of intubation and higher mortality risk. Nonetheless, our data suggest that early intubation timing is critical for survival prognosis because early intubation is associated with higher survival rates than late intubation [18].

Elsewhere, our data showed that for much of the older female population 56.6% (Table 2), the primary mortality risk factors within the 1^st^ week were also diabetes, CKD, and pneumonia (Table 3a). These findings align with other patients afflicted with diabetes who are hospitalized with COVID-19 because these populations have been shown to have a higher risk in developing cardiovascular complications, such as CVD, coronary heart disease, stroke, and increased mortality, than those without COVID-19, highlighting the importance of the role of diabetes for better strategies in patients with diabetes with COVID-19 [19]. Could the increased likelihood of elevated ORs in patients who stay in the ICU for more than 15 days potentially be understood by analyzing the relevant biochemical parameters present in these patients? Unfortunately, the data source we relied on lacks this vital information [8]. Consequently, we were unable to investigate the underlying biochemical factors in this specific long-stay population. It is important to note that our data source also does not include crucial information about the type and stage of disease among the sampled populations.

## Conclusion

The present study focused on hospitalized women with COVID-19 in Mexico City and revealed significant findings regarding comorbidities and mortality rates. Comorbidities such as pneumonia, hypertension, diabetes, obesity, and intubation were prevalent in both age-stratified groups of women, with older women showing higher rates. Not only did they exhibit a higher prevalence of these comorbidities but they also had higher odds of being admitted to the ICU and staying for longer periods.

Contrary to expectations, although obesity and hypertension ranked among the top comorbidities, they did not show a statistically significant impact on the prevalence of fatalities. However, chronic health conditions, such as diabetes, CKD, and pneumonia, emerged as significant mortality risk factors in patients who died within the 1^st^ week after hospital admission. In the 2^nd^ week, conditions shifted to include immunosuppression, ICU transfer, and intubation, with the latter emerging as a significant mortality risk, with the odds increasing as time progressed.

Intubation, particularly, in patients in the ICU, remained an important mortality risk factor, with ORs in deceased patients progressively increasing every day, reaching dangerous levels beyond day 15. This trend underscores the association between delayed intubation and higher mortality risk, particularly, after the 2^nd^ week of admission to critical care units.

Our results underscore the importance of understanding comorbidities and their impact on COVID-19 management, especially in older women, to develop more effective strategies for reducing mortality risks. These findings are critical for health care professionals and policymakers alike.

## Declaration of competing interest

The authors have no competing interests to declare.
